# Tracing the evolution of cardiology: Pioneering discoveries and paradigm shifts in cardiovascular care

**DOI:** 10.1016/j.ahjo.2026.100792

**Published:** 2026-04-29

**Authors:** Krunal Shukla, Austin Natalie, Matthew Peters, Natalie Bracewell

**Affiliations:** a Department of Internal Medicine, University of Florida, Gainesville, FL, United States of America; b Department of Cardiology, University of Florida, Gainesville, FL, United States of America

**Keywords:** Cardiac testing, Cardiac catheters, Heart failure, Cardiac imaging

## Abstract

Cardiology has undergone an impressive transformation from the initial anatomical observations of William Harvey to the current data-driven precision medicine. This review highlights the major milestones that have defined the field's evolution along key discoveries, technologies, and clinical trials that have led to evidence-based cardiovascular care. Cardiovascular disease is the number one cause of death worldwide. Procedural breakthroughs such as coronary artery bypass grafting, percutaneous coronary intervention, and transcatheter valve replacement have transformed management of major adverse cardiovascular events. Simultaneously, landmark clinical trials have led to evidence-based therapies for hypertension, heart failure, and ischemic heart disease. Through synthesizing historical progress and contemporary innovations, this review underscores the transition of cardiology into a discipline that both prolongs and improves quality of life. Through appreciating important milestones, this review provides perspective for the next frontiers in cardiology: integrating artificial intelligence, promoting precision medicine, and genomics.

## Highlights

• Cardiology evolved from foundational physiologic observation to technologically driven precision medicine.
• Breakthroughs in imaging, procedures, and pharmacotherapy have progressively reduced cardiovascular morbidity and mortality
• Landmark trials and the work of professional societies have created evidence-based, guideline-directed cardiovascular care.
• Procedural innovations including CABG, PCI, and transcatheter valve interventions have transformed management of heart disease.

## 1 Introduction

The origination of cardiology can be traced back to the works of William Harvey, a British physician in the early 17th century whose discoveries laid the foundation for early cardiovascular medicine. While it is speculated that the heart was known to be of critical importance to the human body prior to this, it was Harvey's publication of *Exercitatio Anatomica de Motu Cordis et Sanguinis in Animalibus (On the Motion of the Heart and Blood in Animals)* in 1628, which explained that circulation is a closed loop system of continuous blood flow with the heart functioning as a pump [1]. This conclusion transformed understanding of human physiology at the time and stimulated interest in cardiology as a scientific discipline.

Over a century later, a French physician named Rene Theophile Hyacinthe Laënnec sought a more effective means for heart sound auscultation. In 1816, he tightly rolled a piece of paper into a tube to listen to the heart sounds of a patient [2]. He discovered that heart sounds could be better auscultated using this acoustic technique rather than by placing an ear directly to the patient's chest and later developed a hollow wooden tube in 1819. This initial model of the stethoscope was refined several times throughout the 19th and 20th centuries into the binaural model that is widely used today [2].

**Fig. 1:**
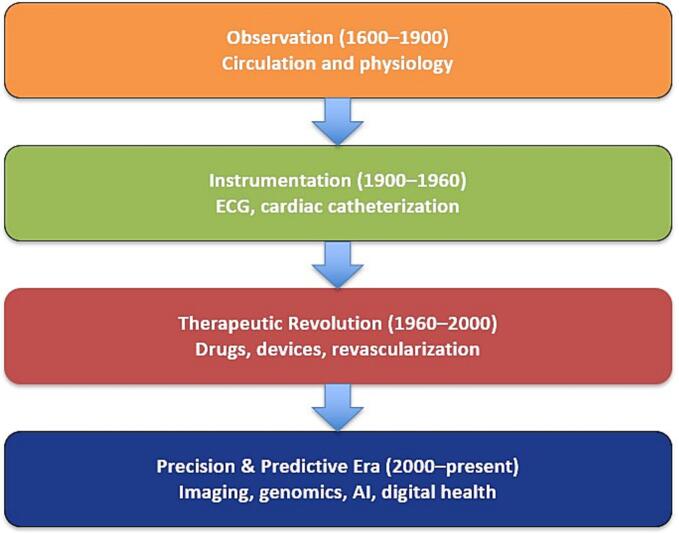
Evolution of cardiology: from observation to precision medicine. This flow diagram depicts the progression of cardiology from early physiologic discovery to modern precision care.

Together, Harvey's discovery of circulation and Laënnec's invention of the stethoscope represent the birth of cardiology as a scientific specialty. This field has since become a constantly evolving practice driven by advances in imaging modalities, pharmacotherapy, treatment approach strategies and interventional technology (Fig. 1). The following discussion traces the evolution of cardiology from the early inventions highlighted above to the increasingly precise and complex field that it is today.

## 2 The Golden Decade

Between the late 18th and mid-19th centuries, important discoveries began to illuminate the electrical mechanisms of cardiac function. Dr. Luigi Galvani demonstrated that an electrical current can be elicited from skeletal muscle contractions, and Dr. Carlo Matteucci then discovered that a frog's heartbeat is accompanied by electrical currents [3]. Nearing the late 19th century, August Waller used a capillary electrometer and electrodes placed on the chest and back of a human to demonstrate the electrical activity preceding a ventricular contraction [3].

In 1903, it was a Dutch physician and physiologist named Dr. Willem Einthoven who furthered Waller's work into the development of a string galvanometer, a 600-pound machine designed to capture the heart's electrical current using a magnetic field [3], [4]. This innovation produced the first electrocardiogram (ECG), ushering in a new era of cardiovascular diagnostics. Einthoven designed the electrodes by immersing the extremities in cylinders of electrolyte solution, ultimately developing the three leads known as Einthoven's triangle, a concept still used to date [3].

Concurrently, other advancements were shaping cardiology's early evolution. In 1895, a German physicist named Wilhelm Roentgen accidentally discovered a new form of radiation that could pass through various materials [4]. He named this “X-ray”, with the ‘X’ representing something unknown [4]. This invention, commonly referred to as the birthplace of radiology, revolutionized medicine by providing the means to non-invasively visualize internal anatomy.

Progress in circulatory understanding followed shortly after. In 1896, an Italian physician and pediatrician named Scipione Riva-Rocci who developed an inflatable cuff to wrap around the upper arm and attached it to a mercury manometer [4]. He noted the absence of a radial pulse with continued cuff inflation. It was then Nikolai Korotkov, a Russian surgeon, who placed a stethoscope on the brachial artery while using Riva-Rocci's invention in 1905 [4]. He noted the sound of blood rushing through the brachial artery when deflating the cuff from supra-systolic pressures as well as the absence of sound after deflating the cuff further [4]. These sounds, coined Korotkov sounds, represented the discovery of systolic and diastolic blood pressures.

The convergence of these discoveries between 1895 and 1905 marked a period commonly referred to as the “Golden Decade” of cardiology. For the first time, physicians possessed the tools to record heart rhythms, measure arterial pressures, and even visualize the heart. These capabilities allowed cardiology to develop into a measurable science and stimulated a new interest in the field, leading to a growing number of ‘heart specialists’, or cardiologists.

## 3 Evolution of cardiac imaging

This section explores the evolution of cardiac imaging. Table 1 highlights key milestones in the development of cardiac imaging.

**Table 1:** Evolution of cardiac imaging.

Modality	First clinical use	Major indications
Cardiac catheterization	Discovered in 1929, integration into clinical practice in early 1940s	Diagnosis and management of coronary artery disease and acute coronary syndrome
Master two-step electrocardiogram	1930s	Assess latent coronary artery disease
Bicycle/treadmill stress test	1950s	Assess cardiovascular function under controlled exercise conditions
Echocardiogram	1950s	Cardiac structure and function assessment
Nuclear stress test	1970s	Assessment of myocardial perfusion and viability
Cardiac computed tomography	1990s	Quantifying coronary artery calcification and assessing cardiac structure and function
Cardiac MRI	1980s	Tissue characterization, function, fibrosis

### 3.1 Cardiac catheterization

In the United States, approximately 1 million cardiac catheterizations are performed annually to diagnose and treat patients with a high probability of coronary heart disease and other associated conditions [5]. Since its introduction by Werner Forssman in 1929, the procedure has rapidly evolved and expanded to include coronary, peripheral, vascular, and structural heart procedures [5]. After Forssman's initial experiment, cardiac catheterization was integrated into clinical practice in the early 1940s through the efforts of Andre Courn and and Dickinson Richards. Following their efforts, in October 1958 Mason Sones further expanded the utility of the procedure through the introduction of selective coronary angiography by selectively injecting contrast media into the right coronary artery while attempting to perform an aortogram [6]. In 1967, Melvin Judkins described his novel approach to coronary angiography via a transfemoral approach, an advancement beyond Sones' approach that required brachial arteriotomy. Furthermore, Judkins' use of pre-bent catheters “preshaped into right and left configurations” revolutionized cardiac catheterization [7]. Once deemed impossible, advances in coronary angiography enabled direct, invasive assessment of cardiac anatomy, physiology, and pathology. For example, in 1960, Harold Dodge utilized biplane angiography to determine left ventricular volumes, finding validation from postmortem heart measurements [8]. These advances in coronary angiography ultimately lead to the advent of percutaneous coronary intervention (PCI), which has become the most frequently performed coronary revascularization procedure [9]. The origins in percutaneous coronary interventions trace their roots back to Zurich in 1977 when Andreas Grüntzig performed the first balloon coronary angioplasty after first testing his design on peripheral interventions [10]. In 1994, the first FDA approved balloon-expandable stent, an adjunct to balloon angioplasty, was approved, pioneered by Julio Palmaz and Richard Schatz [11].

### 3.2 Master two-step ECG

The Master two-step ECG, discovered by Arthur Master in 1920s, provided one of the earliest standardized exercise stress tests to detect latent coronary artery disease (CAD). Through requiring patients to perform simple exercises, it allowed clinicians to unmask ischemic changes on ECG that didn't occur at rest. Thus, in the pre-imaging era, it improved the sensitivity of noninvasive CAD screening [12]. Despite its limitations, it was a pivotal innovation that led to the foundation of exercise-induced ECG stress testing, which is still a cornerstone of noninvasive cardiac evaluation [13].

### 3.3 Bicycle stress test

In the early 1950s, the bicycle stress test enabled standardized, reproducible, and safe assessment of cardiovascular function under controlled exercise conditions. This was formalized in 1963 when Robert A. Bruce published a multistage treadmill test which has since led to him being widely credited with developing the first standardized protocol for the stress test. The Bruce protocol, largely exclusive a treadmill exercise test, is still utilized in clinical practice today. [14]. Bicycle stress testing is used more in Europe than the United States, perhaps due to the increased use of transporation in Europe [15]. These advances are especially valuable for patients with orthopedic and neurological conditions as they provide precise control over workload increments and allow imaging at peak stress. Its versatility and adaptability in helping with simple detection of CAD to comprehensive cardiovascular risk assessment and prognostication has made it an important tool in clinical and preventive cardiology [16].

### 3.4 Echocardiogram

In 1953, Inge Edler and Hellmuth Hertz invented echocardiogram in Lund, Sweden. Echocardiography fundamentally transformed the field of cardiac imaging as it enabled noninvasive, real-time visualization of cardiac anatomy and function. Today, it is the most widely used and essential imaging modality, providing critical diagnostic and prognostic information for a broad spectrum of cardiovascular diseases [17], [18].

### 3.5 Treadmill stress test

In the early 1950s, Dr. Robert A. Bruce at the University of Washington invented the treadmill stress test known as the Bruce protocol. Its introduction allowed simulation and detection of myocardial ischemia, stratify risk, and guide management decisions in patients with possible or known CAD. Currently, it is integral for diagnosis, prognosis, and therapeutic decision-making in a wide range of cardiac conditions [19], [20], [21].

### 3.6 Nuclear stress test

The nuclear stress test was invented in the early 1970s when thallium-201 started to be used as a radiotracer. Dr. Barry Zaret was one of the early pioneers who helped to establish its use in evaluating coronary artery disease. It allowed noninvasive assessment of myocardial perfusion and viability, which was previously only possible with an invasive procedure such as cardiac catheterization. Unlike standard exercise ECG, nuclear imaging allows localization of perfusion defects and distinguishes viable and nonviable myocardium, providing prognostic information for future cardiac events. This led to improvement in cardiac risk stratification along with increased accuracy in the diagnosis of coronary artery disease [22], [23], [24].

### 3.7 Cardiac computed tomography

Cardiac computed tomography (CT), invented in the late 1990s, provided a noninvasive, highly accurate method for visualizing coronary artery anatomy, quantifying coronary artery calcification, and assessing cardiac structure and function. For patients presenting with chest pain, it has become a reliable tool for ruling out significant coronary artery disease, reducing the need for invasive coronary angiography. Major professional societies are now endorsing cardiac CT as a first-line diagnostic tool [25], [26].

### 3.8 Cardiac magnetic resonance imaging

Cardiac magnetic resonance imaging (CMR) was invented in the late 1970s and is a non-invasive, radiation-free modality capable of comprehensive, multiparametric assessment of cardiac structure, function, perfusion, and tissue characterization [27]. It is now gold standard for detailed tissue characterization, especially myocardial fibrosis, edema, infiltration, and viability, which has led to better diagnostic accuracy for ischemic and non-ischemic cardiomyopathies and congenital heart disease [28]. CMR also has the ability to differentiate reversible and irreversible myocardial injury, which has significantly impacted therapeutic decision-making and patient outcomes [29].

### 3.9 Magnetocardiography (1990-present) “no touch” analysis of cardiac magnetic fields

Magnetocardiography (MCG) was invented by David Cohen in 1963 [30]. It has provided a noninvasive, contactless method to record the magnetic fields generated by cardiac electrical activity, allowing a unique way for measuring primary cardiac currents and detecting subtle electrophysiological changes seen in ischemia, arrhythmias, and conduction abnormalities [31]. Therefore, it has enabled noninvasive localization of arrhythmogenic foci and accessory pathways, showing potential for better risk stratification for sudden cardiac death [32], [33].

## 4 Early days of inpatient cardiology

In the early days of inpatient cardiology, the most common diagnoses were acute myocardial infarction, life-threatening arrhythmias, heart failure, and cardiogenic shock. Acute myocardial infarction was one of the main reasons for the development of coronary care units, which specialized in rapid resuscitation and arrhythmia management [34], [35]. Over time, the cases broadened to include more complex presentations, but in the earliest era, the central focus was acute myocardial infarction and its complications [36].

### 4.1 The advent of the Coronary Care Unit

Before 1960, patients hospitalized with acute myocardial infarction were treated with bed rest, oxygen, anticoagulants, and sedatives. In 1962, Hughes Day started the first operational Coronary Care Unit (CCU) at Bethany Medical Center. By 1967, there was published data demonstrating that CCU-monitored patients had improved survival [37].

While CCUs reduced in-hospital mortality, the majority of cardiac deaths occurred before patients reached the hospital. J. Frank Pantridge and his registrar John Geddes launched the world's first mobile coronary care unit from the Royal Victoria Hospital [38]. Eventually, their efforts demonstrated that out-of-hospital cardiac arrest was survivable when treated promptly. Pantridge demonstrated the use of portable defibrillator and eventually developed progressively lighter defibrillators [39].

### 4.2 The early days of electrophysiology

In 1967, the concept of programmed electrical stimulation to induce and study arrhythmias was introduced [40]. Dr. Bernard Lown performed the first successful electrical cardioversion in 1962 [41]. He reported successful cardioversion to terminate atrial fibrillation and other supraventricular arrhythmias, including its application in patients with mitral stenosis and in the post-cardiac surgery setting [42], [43]. Melvin Scheinman performed the first catheter ablation in humans, delivering a high-energy DC shock to interrupt AV conduction in a patient with drug-refractory supraventricular tachycardia [44]. This led directly to the development of radiofrequency ablation catheters in the late 1980s, which created precise, targeted lesions using controlled heat [44].

External cardiac pacing was first demonstrated by Paul Zoll in 1952 [45]. Eventually, Ake Senning and Rune Elmqvist performed the first implantation of a fully self-contained pacemaker in Stockholm [45]. Later on in 1980, Myron Weisfeldt and Philip Reed performed the first successful human ICD implantation in a patient with refractory ventricular arrhythmias [46].

### 4.3 Early cardiac research

The initiation for research into the etiology of cardiac disease, the discovery of risk factors such as smoking, hypertension, elevated lipids, and family history occurred after the dramatic rise in CAD mortality in the early to mid-20th century. It prompted epidemiologists and cardiologists to seek underlying causes. This led to large-scale prospective cohort studies, such as the Framingham Heart Study (FHS) by the US Public Health Service and National Heart Institute in 1948. The FHS study collected longitudinal data on various risk factors and eventually identified hypertension, hyperlipidemia, obesity, and smoking as independent risk predictors for coronary artery disease incidence and mortality [47], [48], [49]. This study was also the turning point in cardiology when care shifted from reactive to proactive management of patients [48].

### 4.4 From thrombolysis to primary percutaneous coronary intervention

The GISSI trial in 1986 demonstrated that use of streptokinase led to lower mortality followed by TIMI-1 trial showing that alteplase led to higher coronary reperfusion compared to streptokinase [50], [51]. Eventually, the PAMI trial in 1993 compared primary PCI to thrombolytic therapy. PCI led to lower combined reinfarction and death. In 2003, DANAMI-2 trial demonstrated PCI superiority even when patients required inter-hospital transfer [52].

### 4.5 Advent of professional societies and scientific congresses

The rapid translation of cardiovascular discoveries into clinical practice has depended on professional societies for education, research dissemination, and guideline development.

The American Heart Association (AHA) was founded in 1924. Its focus on secondary prevention in cardiology started in mid-1990s following its first consensus statement on secondary prevention in 1995, which was also endorsed by the American College of Cardiology (ACC). Since then, both the AHA and the ACC have regularly updated their guidelines and scientific statements to reflect emerging evidence associated with secondary prevention, including comprehensive risk factor management for patients with atherosclerotic cardiovascular disease [53], [54], [55].

The ACC was founded in 1949 by thirteen cardiologists led by Franz Groedel and Bruno Kisch with its first scientific session in New York City in 1951 [56]. Around the same time, the European Society of Cardiology (ESC) was founded. In 1950, the first meeting was held [57]. Together, these organizations built the foundation through which cardiovascular knowledge is validated and translated into practice.

### 4.6 Direct oral anticoagulants

Direct Oral Anticoagulants (DOACs) have transformed stroke prevention in atrial fibrillation. In 2011, two landmark trials established this new drug class as a viable alternative to warfarin: ROCKET-AF demonstrated that rivaroxaban was noninferior to warfarin [58], [59], while ARISTOTLE showed that apixaban was superior, with significant reductions in stroke, bleeding, and mortality [59]. A subsequent meta-analysis confirmed that DOACs collectively reduced stroke, all-cause mortality, and intracranial hemorrhage compared with warfarin [60].

### 4.7 Statins

Statins were first introduced into clinical practice in 1987 with lovastatin being the initial agent in this class [61]. The introduction of statins transformed cardiology by shifting care from symptomatic management of CAD to aggressive risk factor modification and prevention. The Scandinavian Simvastatin Survival Study (4S) in 1994 was a landmark trial that established that cholesterol-lowering with statins was safe and should be standard therapy for patients with coronary artery disease [62].

Through more robust evidence from clinical trials, including LIPID, CARE, and WOSCOPS, statins became the first-line therapy for both primary and secondary prevention of atherosclerotic cardiovascular disease (ASCVD) [63], [64], [65].

### 4.8 Angiotensin-converting enzyme inhibitors

Angiotensin-converting enzyme (ACE) inhibitors were first introduced into clinical practice in 1981. Within a short time, they became a foundational therapy for hypertension, heart failure, and post-myocardial infarction (MI) management. The CONSENSUS trial in 1987 showed 40% reduction in mortality in severe heart failure with enalapril through afterload reduction, prevention of adverse ventricular remodeling, and attenuation of neurohormonal activation [66], [67].

### 4.9 Angiotensin receptor blockers

The introduction of angiotensin receptor blockers (ARBs) transformed cardiology by providing a highly effective, well-tolerated alternative to ACE inhibitors for the management of hypertension, heart failure, and cardiovascular risk, especially in patients intolerant to ACE inhibitors. Large randomized trials recommend ARBs as first-line agents for patients who cannot tolerate ACE inhibitors, fundamentally expanding therapeutic options and improving outcomes for millions of patients [68], [69].

### 4.10 Guideline-directed medical therapy

Guideline-directed medical therapy (GDMT) has fundamentally transformed cardiology by standardizing care, improving patient outcomes, and driving the adoption of evidence-based treatments. In heart failure, the evolution of GDMT has introduced multiple drug classes that target key pathophysiological pathways, resulting in significant reductions in morbidity and mortality [70], [71]. This has led to a shift in cardiology toward a model of care that is evidence-based, standardized, and focused on optimizing patient outcomes [72], [73].

## 5 Outpatient management of cardiac patients

Ambulatory cardiology care has been evolving since at least the early 2010s, with intensified outpatient programs and structured disease management shown to reduce mortality and hospitalizations in chronic cardiac conditions by 2015–2016 [74]. However, the most dramatic and rapid expansion of ambulatory management occurred during the COVID-19 pandemic, when telehealth visits replaced in-person encounters for up to 30% of all US ambulatory visits, including cardiology, and remote monitoring became routine for many patients [75], [76]. This shift enabled earlier detection of problems and more timely interventions, especially for heart failure, arrhythmias, and hypertension, using wearable devices, home monitoring, and virtual visits [77], [78], [79].

The shift toward active ambulatory management in cardiology directly facilitated the adoption of same-day discharge (SDD) after PCI for uncomplicated acute myocardial infarction (AMI) by leveraging advances in patient selection, procedural safety, and structured post discharge follow-up. Ambulatory pathways, including virtual and telephone follow-up, have enabled earlier identification of complications and optimization of secondary prevention, making SDD both feasible and safe for low-risk patients [80], [81].

## 6 The future of cardiology

Cardiovascular disease (CVD) remains the leading cause of mortality globally despite advancements in diagnosis and treatment [82]. While prior decades in cardiology have been defined by discoveries with established clinical benefit, the future of the field is characterized by rapidly evolving areas of research and innovation where long-term impact is still being defined. As such, the future direction of cardiology is better elucidated through broad domains of innovation rather than specific emerging therapies.

One significant area of ongoing development is genetics and precision medicine. Improved understanding of the genetic contributions to cardiovascular disease has allowed for more accurate identification of inherited conditions and refined cardiovascular risk stratification. As genetic characterization technologies continue to develop, they hold promise in enabling better individualized approaches to prevention and treatment.

A second emerging domain involves immunologic and molecular-targeted therapies. Growing recognition of the role of inflammation and immune pathways in cardiovascular disease has led to research into potential therapies directed at these mechanisms [83]. Early studies suggest potential benefit in these areas, while the long-term clinical impact has yet to be established.

A third important area of progress is the expansion of patient-centered diagnostics, including wearable and implantable technologies. Continuous monitoring through these devices offers the potential for earlier disease detection as well as improved longitudinal management [84]. While these tools are becoming more reliable for clinical integration, their impact on long-term patient outcomes remain under investigation.

In parallel, advances in imaging techniques, data science, and artificial intelligence are expected to further augment diagnostic precision and support clinical decision-making. Collectively, these developments also represent a shift toward more personalized, proactive, and data-driven cardiovascular care.

Many of these emerging technologies and therapies have not yet demonstrated long-term clinical benefit. However, like the historical advances described above, there is potential for substantial impact not only on diagnostics and treatment for the individualized patient, but with a potential global impact for the prevention and management of heart disease. Their role in routine clinical practice will depend on the results of ongoing studies and their ability to meaningfully improve patient outcomes.

## 7 Conclusion

Over the past 50 years, cardiology has undergone a remarkable transformation driven by research, evidence-based medicine, and advances in pathophysiologic understanding. In the 1970s and early 1980s, cardiology was largely diagnostic-centered, relying heavily on physical exam, basic EKG interpretation, and limited imaging. CABG was a major breakthrough, followed by the rapid development of PCI, turning ACS from a highly fatal event into a relatively treatable condition.

In the last two decades, cardiology has continued to move toward precision and minimally invasive interventions. Imaging has also advanced from simple echocardiography to 3D echocardiogram, cardiac MRI, and CT angiography, allowing the visualization of fine anatomic and functional details. Cardiology is and will continue to be an exciting field as it incorporates genomics, AI-driven diagnostics, and device-based therapeutics, continuing to shift from reactive treatment to prevention.

## Ethics in publishing statement

I testify on behalf of all co-authors that our article submitted followed ethical principles in publishing.

## CRediT authorship contribution statement

**Krunal Shukla:** Writing – review & editing, Writing – original draft, Visualization, Conceptualization. **Austin Natalie:** Writing – review & editing, Writing – original draft, Conceptualization. **Matthew Peters:** Writing – review & editing, Writing – original draft. **Natalie Bracewell:** Writing – review & editing, Writing – original draft, Supervision, Conceptualization.

## Declaration of competing interest

All authors have no disclosures or conflicts of interest to declare.

## References

[1] Harvey W. (1962). On the Motion of the Heart and Blood in Animals (Willis's trans., rev. and edited). [Internet].

[2] Roguin A. (2006 Sep). Rene Theophile Hyacinthe Laënnec (1781-1826): the man behind the stethoscope. Clin. Med. Res..

[3] AlGhatrif M., Lindsay J. (2012 Apr 30). A brief review: history to understand fundamentals of electrocardiography. J. Community Hosp. Intern. Med. Perspect..

[4] Braunwald E. (2021 May 1). The birth of cardiology: the golden decade. Eur. Heart J..

[5] Bangalore S., Barsness G.W., Dangas G.D., Kern M.J., Rao S.V., Shore-Lesserson L. (2021 Aug 3). Evidence-based practices in the cardiac catheterization laboratory: a scientific statement from the American Heart Association. Circulation.

[6] Bourassa M.G. (2005 Oct). The history of cardiac catheterization. Can. J. Cardiol..

[7] Judkins M.P. (1967 Nov). Selective coronary arteriography. I. A percutaneous transfemoral technic. Radiology.

[8] Dodge H.T., Sandler H., Ballew D.W., Lord J.D. (1960 Nov). The use of biplane angiocardigraphy for the measurement of left ventricular volume in man. Am. Heart J..

[9] Holmes D.R., Williams D.O. (2008 Aug). Catheter-based treatment of coronary artery disease: past, present, and future. Circ. Cardiovasc. Interv..

[10] Barton M., Grüntzig J., Husmann M., Rösch J. (2014 Dec 29). Balloon angioplasty - the legacy of Andreas Grüntzig, M.D. (1939–1985). Front. Cardiovasc. Med..

[11] Williams D.O. (1996 Feb). Dressing up the Palmaz-Schatz stent. Circulation.

[12] Teichholz L.E., Cohn P.F., Gorlin R. (1975 Apr). The omnicardiogram: new approach to detection of heart disease in patients with a normal resting electrocardiogram. Am. J. Cardiol..

[13] Kadish A.H., Buxton A.E., Kennedy H.L., Knight B.P., Mason J.W., Schuger C.D. (2001 Dec). ACC/AHA clinical competence statement on electrocardiography and ambulatory electrocardiography. A report of the ACC/AHA/ACP-ASIM Task Force on Clinical Competence (ACC/AHA Committee to Develop a Clinical Competence Statement on Electrocardiography and Ambulatory Electrocardiography). J. Am. Coll. Cardiol..

[14] Bruce R.A., Blackmon J.R., Jones J.W., Strait G. (2004 Jul). Exercising testing in adult normal subjects and cardiac patients. Ann. Noninvasive Electrocardiol..

[15] Moss A.J. (2004 Jul 5). Exercise testing. Ann. Noninvasive Electrocardiol..

[16] Löllgen H., Leyk D. (2018 Jun 15). Exercise testing in sports medicine. Dtsch. Arztebl. Int..

[17] Fraser A.G., Monaghan M.J., van der Steen A.F.W., Sutherland G.R. (2022 Aug 22). A concise history of echocardiography: timeline, pioneers, and landmark publications. Eur. Heart J. Cardiovasc. Imaging.

[18] Wiegers S.E., Ryan T., Arrighi J.A., Brown S.M., Canaday B., Damp J.B. (2019 Jul 23). 2019 ACC/AHA/ASE advanced training statement on echocardiography (revision of the 2003 ACC/AHA clinical competence statement on echocardiography): a report of the ACC competency management committee. J. Am. Coll. Cardiol..

[19] Fletcher G.F., Ades P.A., Kligfield P., Arena R., Balady G.J., Bittner V.A. (2013 Aug 20). Exercise standards for testing and training: a scientific statement from the American Heart Association. Circulation.

[20] Rodgers G.P., Ayanian J.Z., Balady G., Beasley J.W., Brown K.A., Gervino E.V. (2000 Oct). American College of Cardiology/American Heart Association Clinical Competence statement on stress testing: a report of the American College of Cardiology/American Heart Association/American College of Physicians–American Society of Internal Medicine Task Force on Clinical Competence. J. Am. Coll. Cardiol..

[21] Goldschlager N., Selzer A., Cohn K. (1976 Sep). Treadmill stress tests as indicators of presence and severity of coronary artery disease. Ann. Intern. Med..

[22] Yoshinaga K., Manabe O., Tamaki N. (2011 Feb 3). Physiological assessment of myocardial perfusion using nuclear cardiology would enhance coronary artery disease patient care: which imaging modality is best for evaluation of myocardial ischemia? (SPECT-side). Circ. J..

[23] Zaret B.L., Cohen L.S. (1975 Jan). Radionuclides and the patient with coronary artery disease. Am. J. Cardiol..

[24] Strauss H.W. (1976 Nov). Cardiovascular nuclear medicine: a new look at an old problem. Noninvasive approaches to the evaluation of coronary heart disease: new horizons for radiologists lecture. Radiology.

[25] Pontone G., Rossi A., Guglielmo M., Dweck M.R., Gaemperli O., Nieman K. (2022 Mar 22). Clinical applications of cardiac computed tomography: a consensus paper of the European Association of Cardiovascular Imaging-Part II. Eur. Heart J. Cardiovasc. Imaging.

[26] Schroeder S., Achenbach S., Bengel F., Burgstahler C., Cademartiri F., de Feyter P. (2008 Feb). Cardiac computed tomography: indications, applications, limitations, and training requirements: report of a Writing Group deployed by the Working Group Nuclear Cardiology and Cardiac CT of the European Society of Cardiology and the European Council of Nuclear Cardiology. Eur. Heart J..

[27] Arnold J.R., McCann G.P. (2020 Feb). Cardiovascular magnetic resonance: applications and practical considerations for the general cardiologist. Heart.

[28] Rajiah P.S., François C.J., Leiner T. (2023 May). Cardiac MRI: state of the art. Radiology.

[29] Sawlani R.N., Collins J.D. (2016 May). Cardiac MRI and ischemic heart disease: role in diagnosis and risk stratification. Curr. Atheroscler. Rep..

[30] Roth B.J. (2023 Apr 23). Biomagnetism: the first sixty years. Sensors (Basel, Switzerland).

[31] Yamada S., Yamaguchi I. (2005 Jan). Magnetocardiograms in clinical medicine: unique information on cardiac ischemia, arrhythmias, and fetal diagnosis. Intern. Med..

[32] Moshage W., Achenbach S., Göhl K., Weikl A., Bachmann K., Wegener P. (1991 Sep). Biomagnetic localization of ventricular arrhythmias. Radiology.

[33] Weismüller P., Abraham-Fuchs K., Schneider S., Richter P., Kochs M., Hombach V. (1992 May). Magnetocardiographic non-invasive localization of accessory pathways in the Wolff-Parkinson-White syndrome by a multichannel system. Eur. Heart J..

[34] Fuster V. (1999 Dec). 50th anniversary historical article. Myocardial infarction and coronary care units. J. Am. Coll. Cardiol..

[35] Sinha S.S., Geller B.J., Katz J.N., Arslanian-Engoren C., Barnett C.F., Bohula E.A. (2025 Mar 11). Evolution of critical care cardiology: an update on structure, care delivery, training, and research paradigms: a scientific statement from the American Heart Association. Circulation.

[36] Braunwald E. (2012 Apr). The rise of cardiovascular medicine. Eur. Heart J..

[37] Fye W.B. (2011 Oct 25). Resuscitating a circulation abstract to celebrate the 50th anniversary of the coronary care unit concept. Circulation.

[38] Eisenberg M.S., Pantridge J.F., Cobb L.A., Geddes J.S. (1996 Aug 26). The revolution and evolution of prehospital cardiac care. Arch. Intern. Med..

[39] Pantridge J.F., Geddes J.S. (1967 Aug 5). A mobile intensive-care unit in the management of myocardial infarction. Lancet.

[40] Wellens H.J., Schuilenburg R.M., Durrer D. (1972 Aug). Electrical stimulation of the heart in patients with ventricular tachycardia. Circulation.

[41] Cakulev I., Efimov I.R., Waldo A.L. (2009 Oct 20). Cardioversion: past, present, and future. Circulation.

[42] Lown B., Amarasingham R., Neuman J. (1962 Nov 3). New method for terminating cardiac arrhythmias. Use of synchronized capacitor discharge. JAMA.

[43] Lown B., Perlroth M.G., Kaidbey S., Abe T., Harken D.E. (1963 Aug 15). “Cardioversion” of atrial fibrillation. A report on the treatment of 65 episodes in 50 patients. N. Engl. J. Med..

[44] Scheinman M.M., Morady F., Hess D.S., Gonzalez R. (1982 Aug 20). Catheter-induced ablation of the atrioventricular junction to control refractory supraventricular arrhythmias. JAMA J. Am. Med. Assoc..

[45] van Hemel N.M., van der Wall E.E. (2008 Oct). 8 October 1958, D Day for the implantable pacemaker. Neth. Hear. J..

[46] Kastor J.A. (1989 May 1). Michel Mirowski and the automatic implantable defibrillator. Am. J. Cardiol..

[47] Chen G., Levy D. (2016 Oct 1). Contributions of the Framingham Heart Study to the epidemiology of coronary heart disease. JAMA Cardiol..

[48] Nabel E.G., Braunwald E. (2012 Jan 5). A tale of coronary artery disease and myocardial infarction. N. Engl. J. Med..

[49] Keil U. (2000). Coronary artery disease: the role of lipids, hypertension and smoking. Basic Res. Cardiol..

[50] (1986 Feb 22). Effectiveness of intravenous thrombolytic treatment in acute myocardial infarction. Gruppo Italiano per lo Studio della Streptochinasi nell'Infarto Miocardico (GI\SSI). Lancet.

[51] Chesebro J.H., Knatterud G., Roberts R., Borer J., Cohen L.S., Dalen J. (1987 Jul). Thrombolysis in Myocardial Infarction (TIMI) Trial, Phase I: a comparison between intravenous tissue plasminogen activator and intravenous streptokinase. Clinical findings through hospital discharge. Circulation.

[52] Andersen H.R., Nielsen T.T., Rasmussen K., Thuesen L., Kelbaek H., Thayssen P. (2003 Aug 21). A comparison of coronary angioplasty with fibrinolytic therapy in acute myocardial infarction. N. Engl. J. Med..

[53] Smith S.C., Allen J., Blair S.N., Bonow R.O., Brass L.M., Fonarow G.C. (2006 May 16). AHA/ACC guidelines for secondary prevention for patients with coronary and other atherosclerotic vascular disease: 2006 update: endorsed by the National Heart, Lung, and Blood Institute. Circulation.

[54] Fleg J.L., Forman D.E., Berra K., Bittner V., Blumenthal J.A., Chen M.A. (2013 Nov 26). Secondary prevention of atherosclerotic cardiovascular disease in older adults: a scientific statement from the American Heart Association. Circulation.

[55] Leon A.S., Franklin B.A., Costa F., Balady G.J., Berra K.A., Stewart K.J. (2005 Jan 25). Cardiac rehabilitation and secondary prevention of coronary heart disease: an American Heart Association scientific statement from the Council on Clinical Cardiology (Subcommittee on Exercise, Cardiac Rehabilitation, and Prevention) and the Council on Nutrition, Physical Activity, and Metabolism (Subcommittee on Physical Activity), in collaboration with the American association of Cardiovascular and Pulmonary rehabilitation. Circulation.

[56] Schlepper M. (1988 Aug). Franz Groedel, Bruno Kisch and the founding of the American College of Cardiology. J. Am. Coll. Cardiol..

[57] Berry D. (2009 Apr). Development of the ECG from string to wireless. Eur. Heart J..

[58] Patel M.R., Mahaffey K.W., Garg J., Pan G., Singer D.E., Hacke W. (2011 Sep 8). Rivaroxaban versus warfarin in nonvalvular atrial fibrillation. N. Engl. J. Med..

[59] Granger C.B., Alexander J.H., McMurray J.J.V., Lopes R.D., Hylek E.M., Hanna M. (2011 Sep 15). Apixaban versus warfarin in patients with atrial fibrillation. N. Engl. J. Med..

[60] Ruff C.T., Giugliano R.P., Braunwald E., Hoffman E.B., Deenadayalu N., Ezekowitz M.D. (2014 Mar 15). Comparison of the efficacy and safety of new oral anticoagulants with warfarin in patients with atrial fibrillation: a meta-analysis of randomised trials. Lancet.

[61] Tobert J.A. (2003 Jul). Lovastatin and beyond: the history of the HMG-CoA reductase inhibitors. Nat. Rev. Drug Discov..

[62] 4S Study Group (1994 Nov 19). Randomised trial of cholesterol lowering in 4444 patients with coronary heart disease: the Scandinavian Simvastatin Survival Study (4S). Lancet.

[63] Liao J.K. (2002 Nov). Beyond lipid lowering: the role of statins in vascular protection. Int. J. Cardiol..

[64] Vaughan C.J., Gotto A.M., Basson C.T. (2000 Jan). The evolving role of statins in the management of atherosclerosis. J. Am. Coll. Cardiol..

[65] Doggrell S.A. (2001 Sep). Statins in the 21st century: end of the simple story?. Expert Opin. Investig. Drugs.

[66] Harrington J., Petrie M.C., Anker S.D., Bhatt D.L., Jones W.S., Udell J.A. (2022 Oct 1). Evaluating the application of chronic heart failure therapies and developing treatments in individuals with recent myocardial infarction: a review. JAMA Cardiol..

[67] Khalil M.E., Basher A.W., Brown E.J., Alhaddad I.A. (2001 Jun 1). A remarkable medical story: benefits of angiotensin-converting enzyme inhibitors in cardiac patients. J. Am. Coll. Cardiol..

[68] Heidenreich P.A., Bozkurt B., Aguilar D., Allen L.A., Byun J.J., Colvin M.M. (2022 May 3). 2022 AHA/ACC/HFSA guideline for the management of heart failure. JACC.

[69] Ram C.V.S. (2008 Aug). Angiotensin receptor blockers: current status and future prospects. Am. J. Med..

[70] Murphy S.P., Ibrahim N.E., Januzzi J.L. (2020 Aug 4). Heart failure with reduced ejection fraction: a review. JAMA J. Am. Med. Assoc..

[71] Velez M. (2023 Mar 1). Advances in contemporary medical management to treat patients with heart failure. Curr. Opin. Cardiol..

[72] Goldfarb M.J., Saylor M.A., Bozkurt B., Code J., Di Palo K.E., Durante A. (2024 May 14). Patient-centered adult cardiovascular care: a scientific statement from the American Heart Association. Circulation.

[73] Maron D.J., Newman J.D., Anthopolos R., Lu Y., Stevens S., Boden W.E. (2025 Apr 1). Guideline-directed medical therapy and outcomes in the ISCHEMIA trial. J. Am. Coll. Cardiol..

[74] Sawicki O.A., Mueller A., Glushan A., Breitkreuz T., Wicke F.S., Karimova K. (2020 Sep 7). Intensified ambulatory cardiology care: effects on mortality and hospitalisation-a comparative observational study. Sci. Rep..

[75] Takahashi E.A., Schwamm L.H., Adeoye O.M., Alabi O., Jahangir E., Misra S. (2022 Dec 20). An overview of telehealth in the management of cardiovascular disease: a scientific statement from the American Heart Association. Circulation.

[76] Yuan N., Pevnick J.M., Botting P.G., Elad Y., Miller S.J., Cheng S. (2021 Apr 1). Patient use and clinical practice patterns of remote cardiology clinic visits in the era of COVID-19. JAMA Netw. Open.

[77] Sana F., Isselbacher E.M., Singh J.P., Heist E.K., Pathik B., Armoundas A.A. (2020 Apr 7). Wearable devices for ambulatory cardiac monitoring: JACC state-of-the-art review. J. Am. Coll. Cardiol..

[78] Spatz E.S., Ginsburg G.S., Rumsfeld J.S., Turakhia M.P. (2024 Jan 25). Wearable digital health technologies for monitoring in cardiovascular medicine. N. Engl. J. Med..

[79] Kennel P.J., Rosenblum H., Axsom K.M., Alishetti S., Brener M., Horn E. (2022 May 1). Remote cardiac monitoring in patients with heart failure: a review. JAMA Cardiol..

[80] Rathod K.S., Comer K., Casey-Gillman O., Moore L., Antoniou S., Fhadil S. (2025 Jun 23). Cost-effectiveness of early discharge (\textless48 hours) for low-risk patients following PPCI for STEMI. JACC Cardiovasc. Interv..

[81] Rathod K.S., Comer K., Casey-Gillman O., Moore L., Mills G., Ferguson G. (2021 Dec 21). Early hospital discharge following PCI for patients with STEMI. J. Am. Coll. Cardiol..

[82] (2025). Cardiovascular diseases [WEBSITE] [Internet]. https://www.who.int/health-topics/cardiovascular-diseases?utm\_source=chatgpt.com\#tab=tab\_1.

[83] Ajoolabady A., Pratico D., Lin L., Mantzoros C.S., Bahijri S., Tuomilehto J. (2024 Nov 11). Inflammation in atherosclerosis: pathophysiology and mechanisms. Cell Death Dis..

[84] Armoundas A.A., Ahmad F.S., Bennett D.A., Chung M.K., Davis L.L., Dunn J. (2024 Jun). Data interoperability for ambulatory monitoring of cardiovascular disease: a scientific statement from the American Heart Association. Circ. Genom. Precis. Med..

